# Immune cells in cardiac repair and regeneration

**DOI:** 10.1242/dev.199906

**Published:** 2022-05-03

**Authors:** Filipa C. Simões, Paul R. Riley

**Affiliations:** 1 MRC Weatherall Institute of Molecular Medicine, Radcliffe Department of Medicine, University of Oxford, Oxford, Oxfordshire OX3 9DS, UK; 2 Institute of Developmental and Regenerative Medicine, Old Road Campus, Oxford, Oxfordshire OX3 7DQ, UK; 3 Department of Physiology, Anatomy and Genetics, University of Oxford, Oxford, Oxfordshire OX1 3PT, UK

**Keywords:** Cardiac, Immune system, Muscle, Regeneration

## Abstract

The immune system is fundamental to tissue homeostasis and is the first line of defense following infection, injury or disease. In the damaged heart, large numbers of immune cells are recruited to the site of injury. These cells play an integral part in both repair by scar formation and the initiation of tissue regeneration. They initially assume inflammatory phenotypes, releasing pro-inflammatory cytokines and removing dead and dying tissue, before entering a reparative stage, replacing dead muscle tissue with a non-contractile scar. In this Review, we present an overview of the innate and adaptive immune response to heart injury. We explore the kinetics of immune cell mobilization following cardiac injury and how the different innate and adaptive immune cells interact with one another and with the damaged tissue. We draw on key findings from regenerative models, providing insight into how to support a robust immune response permissible for cardiac regeneration. Finally, we consider how the latest technological developments can offer opportunities for a deeper and unbiased functional understanding of the immune response to heart disease, highlighting the importance of such knowledge as the basis for promoting regeneration following cardiac injury in human patients.

## Abstract (precis)

**Summary:** This Review presents the innate and adaptive immune responses to heart injury in regenerative and non-regenerative organisms to uncover an immune response permissible for cardiac regeneration.

## Introduction

**Fig. 1.**
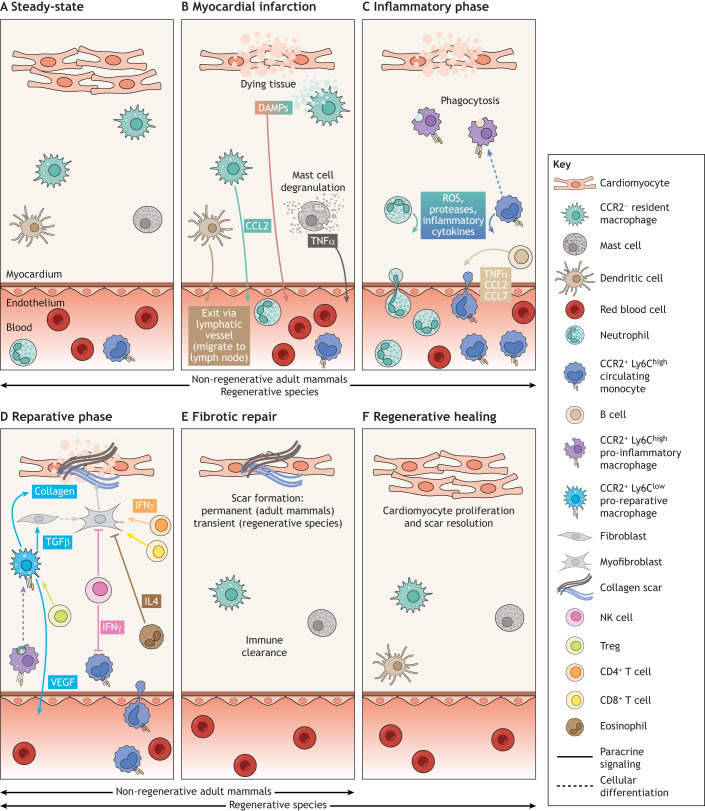
**Immune response to myocardial infarction.** (A) Before injury, CCR2^−^ resident macrophages, mast cells and dendritic cells reside in the heart. (B) Following injury, an orchestrated cellular and molecular cascade takes place in the damaged heart, including the release of damage-associated molecular patterns (DAMPs) from dying cardiomyocytes and resident macrophages, and mast cell degranulation. (C) This activity triggers an inflammatory phase during which infiltrating innate immune cells (such as neutrophils and CCR2^+^ Ly6C^high^ monocytes) are recruited to the site to clear debris. (D) Pro-inflammatory CCR2^+^ Ly6C^high^ monocyte-derived macrophages phagocytose neutrophils to resolve inflammation, leading to an increase in production of anti-inflammatory and pro-fibrotic cytokines (e.g. IL10 and TGFβ) and subsequent downregulation of pro-inflammatory cytokines (e.g. IL1β and TNFα). CCR2^+^ Ly6C^high^ monocyte-derived macrophages then differentiate *in situ* into reparative CCR2^+^ Ly6C^low^ macrophages, via a nuclear receptor subfamily 4 group A member 1 (NR4A1)-dependent transcriptional program and, consequently, inflammation is superseded by a reparative stage. CCR2^+^ Ly6C^low^ monocyte-derived macrophages promote deposition of a fibrotic scar by directly secreting collagen and by inducing myofibroblast formation. (E) The newly formed collagen scar is permanent in adult mammals, leading to fibrotic repair of the heart. (F) However, in regenerative organisms the scar formed is transient in nature, being totally replaced by new cardiac muscle tissue.

The damage caused by a heart attack (myocardial infarction, MI) leads to a permanent loss of cardiac tissue in adult mammals (Sadek and Olson, 2020). MI is often a consequence of coronary artery occlusion due to atherosclerotic plaque rupture in the arterial wall of the heart (Frangogiannis, 2015). This event results in permanent or transient reduced blood flow and ensuing ischemia and low-nutrient-induced tissue injury. Associated cardiomyocyte death activates tissue-resident immune and non-immune cells to produce pro-inflammatory cytokines and chemokines, leading to the recruitment of large numbers of circulating immune cells to the heart (Frangogiannis and Entman, 2005). The immune response is under tight spatiotemporal regulation, with neutrophils and macrophages among the first cells recruited to the site of injury (Fig. 1A,B), followed by activation of the adaptive immune response, including B and T cells (Fig. 1C,D). Immune cells initially assume inflammatory phenotypes, characterized by increased numbers of neutrophil and macrophage populations, which are key in orchestrating the various steps of the post-MI healing process. During the final reparative phase, immune cells promote heart repair by activation of resident fibroblasts and promotion of endothelial cell proliferation (Fig. 1D) (Swirski and Nahrendorf, 2018). The dead muscle tissue is replaced with a non-contractile permanent scar through fibrosis that compromises function, leading to pathological remodeling and ultimately heart failure (Swirski and Nahrendorf, 2018) (Fig. 1E). Therefore, striking a fine balance between a pro-inflammatory versus a pro-fibrotic response is poised to assist the development of immunomodulation therapies that will enhance cardiac repair and offer the potential to invoke enhanced tissue restoration. A key feature to successfully achieve this goal is to fully understand the cellular communication occurring between the many different immune and non-immune cell types that populate the damaged heart. Intercellular signaling after injury modulates the kinetics of immune responses, ultimately establishing a local environment conducive for preserved cardiac morphology and function. Insights into immune responses that specifically favor a regenerative outcome post-MI could inform on how to optimize outcomes in the injured human heart.

## Innate immune response to heart injury

After MI, dead cardiomyocytes release damage-associated molecular patterns (DAMPs) that serve as danger signals and trigger mobilization of the first innate immune cell responders (Arslan et al., 2011) (Fig. 1B). The plethora of infiltrating innate immune cells comprise several functional subtypes and phenotypes as described below.

### Mast cells

During the initial stages post-MI, mast cells are important initiators of the immune response (Frangogiannis et al., 1998). After coronary vessel occlusion, blood flow can be re-established to the still viable ischemic tissue as a way of restoring the levels of oxygen and nutrients. However, inflammatory ‘reperfusion injury’ can occur as a result, of which mast cells are thought to be an important component. Resident cardiac mast cells release pro-inflammatory mediators, such as tumor necrosis factor alpha (TNFα), histamine and mast cell proteases, which initiate a signaling cascade involving neighboring resident macrophages, endothelial cells and subsequently infiltrating neutrophils (Frangogiannis et al., 1998) (Fig. 1B). Cleaved complement proteins, such as C5a, induce neutrophil migration into the site of injury via the CD11b–CD18 complex (Foreman et al., 1996).

### Neutrophils

Neutrophils are initially recruited from the circulating blood and bone marrow to the site of injury by DAMPS and inflammatory signals released by resident cardiac cells, including mast cells (Swirski and Nahrendorf, 2013). Neutrophil pattern recognition receptors, including toll-like receptors (TLRs) and interleukin receptors, bind to DAMPs allowing propagation of inflammatory signaling via cytokine and chemokine production (Bujak et al., 2008). Neutrophils begin infiltrating the damaged heart via permeable blood vessels and rapidly migrate to the infarcted tissue, clearing debris and dead cells through phagocytosis (Daseke et al., 2021) (Fig. 1B). Neutrophil depletion before MI impedes the removal of dead cells, increasing fibrosis and worsening cardiac function (Horckmans et al., 2017).

Neutrophils assume an inflammatory state during their peak response at 1-day post-MI, shifting to an anti-inflammatory and reparative phenotype thereafter (Daseke et al., 2019; Ma et al., 2016; Vafadarnejad et al., 2020). Although neutrophils play important roles in the tissue repair process, they also generate reactive oxygen species (ROS) and secrete proteases, which degrade extracellular matrix (ECM), and exacerbate the post-injury inflammatory response (Frangogiannis, 2012; Ma et al., 2016; Puhl and Steffens, 2019) (Fig. 1C). For this reason, timely neutrophil clearance is crucial to prevent further tissue damage (Lai et al., 2017; Lorchner et al., 2015; Schloss et al., 2016; Xu et al., 2019). Myocardium-infiltrating neutrophils undergo apoptosis and their numbers decrease from 3-days post-MI, almost disappearing by 7 days post-MI (Daseke et al., 2019). They contribute to their own clearance, and therefore the resolution of inflammation, by actively recruiting circulating monocytes and macrophages to the injury site (El Kebir and Filep, 2013) (Fig. 1D). Neutrophils secrete gelatinase-associated lipocalin (NGAL), promoting macrophages to express high levels of myeloid-epithelial-reproductive tyrosine kinase (MertK), a marker of phagocytosis (Horckmans et al., 2017; Scott et al., 2001; Wan et al., 2013). A similar increase in phagocytosis of apoptotic neutrophils is achieved by post-MI treatment with IL4 (Daseke et al., 2020).

### Monocytes and macrophages

Monocytes and macrophages are the predominant immune cell types found within the infarcted myocardium (Epelman et al., 2014; Nahrendorf et al., 2007). The heart, as most tissues, acquires resident macrophage populations early in development, originating from the yolk sac and fetal liver progenitors (Epelman et al., 2014; Perdiguero et al., 2015) (Box 1). These specialized mononuclear phagocytes are partially or entirely replaced over time by circulating monocyte-derived cells that infiltrate the cardiac tissue from the spleen and bone marrow (Molawi et al., 2014; Lee and Ginhoux, 2022). Both tissue-resident and infiltrating-derived macrophages are functionally diverse during steady-state conditions and following injury (Dick et al., 2019; Guilliams et al., 2018; Leid et al., 2016; Perdiguero and Geissmann, 2016; Sansonetti et al., 2020). Expression of CCR2, a chemokine receptor important for migration, distinguishes these populations (Perdiguero and Geissmann, 2016). At steady-state, CCR2^−^ resident cardiac macrophages are either maintained independently of circulating monocytes, if expressing the phosphatidylserine receptor TIMD4^+^, or are partially replaced by CCR2^−^ TIMD4^−^ monocytes, whereas CCR2^+^ resident macrophage subsets are fully replaced by circulating monocytes (Bajpai et al., 2018, 2019; Dick et al., 2019). After MI, embryonic-derived resident CCR2^−^ macrophages decrease in number (Fig. 1B), whereas circulating CCR2^+^ monocytes expressing the surface glycoprotein lymphocyte antigen 6C (Ly6C) are recruited to the site of injury in response to C–C chemokine ligand 2 (CCL2) expression by resident macrophages, cardiomyocytes, mesenchymal stem cells in the bone marrow and peripheral B cells (Dewald et al., 2005; Frangogiannis, 2004; Frangogiannis et al., 2007; Shi et al., 2011; Zouggari et al., 2013) (Fig. 1C). CCR2^+^ Ly6C^+^ monocytes differentiate within the heart into a variety of macrophage subsets (Bajpai et al., 2018, 2019; Dick et al., 2019; Dutta et al., 2015; Epelman et al., 2014; Frantz and Nahrendorf, 2014; Lavine et al., 2014; Nahrendorf et al., 2007). Recruited CCR2^+^ Ly6C^high^ monocyte-derived macrophages initially assume inflammatory phenotypes, releasing pro-inflammatory cytokines, proteolytic enzymes and ROS, to assist neutrophils in the clearance of dead and dying cell debris (Fig. 1C). Subsequently, these pro-inflammatory macrophages act to phagocytose neutrophils (Li et al., 2016) (Fig. 1D), which leads to an increase in production of anti-inflammatory and pro-fibrotic cytokines, such as IL10 and TGFβ, and subsequent downregulation of IL1β and TNFα pro-inflammatory cytokines (Fadok et al., 1998; Voll et al., 1997; Wan et al., 2013). CCR2^+^ Ly6C^high^ monocyte-derived macrophages then differentiate *in situ* into reparative CCR2^+^ Ly6C^low^ macrophages, via a nuclear receptor subfamily 4 group A member 1 (NR4A1)-dependent transcriptional program (Hanna et al., 2011; Hilgendorf et al., 2014). CCR2^+^ Ly6C^low^ accumulate in the injury area, promoting fibrotic heart repair, replacing dead muscle tissue with a permanent scar (Hilgendorf et al., 2014) (Fig. 1D). They modulate ECM turnover by regulating the balance of matrix metalloproteinases and their tissue inhibitors and contribute to scar formation, both by activating cardiac fibroblasts via secretion of TGFβ to become collagen-depositing myofibroblasts (Frangogiannis, 2012; Hilgendorf et al., 2014), and by directly expressing and depositing collagen into the scar (Simoes et al., 2020) (Fig. 1D). Ultimately, the replacement of dead cardiomyocytes with a non-contractile scar stabilizes the injury site, preventing rupture of the ventricle wall (Fig. 1E), but leads to pathological ventricular remodeling and heart failure (Swirski and Nahrendorf, 2018). CCR2^+^ Ly6C^low^ reparative macrophages also secrete growth factors, such as VEGF, to promote cell proliferation and angiogenesis (Frangogiannis, 2012; Hilgendorf et al., 2014) (Fig. 1D). By using a novel experimental approach of coronary ligation in which the pericardium remains intact, a recent study has revealed a population of GATA6^+^ macrophages residing in the pericardial cavity that protect the heart from adverse fibrosis following cardiac injury (Deniset et al., 2019).

**Box 1. The immune system in cardiac development**

Detailed fate-mapping studies have shown that some immune cells populate the heart during development. Particular attention has been given to macrophages, which seed the cardiac tissue from the yolk sac and fetal liver derived progenitors (Epelman et al., 2014; Perdiguero et al., 2015; Lee and Ginhoux, 2022). The epicardium, the outer layer of the heart, is an important signaling hub (Simoes and Riley, 2018) and is involved in the recruitment of yolk sac-derived macrophages to the developing subepicardial space (Stevens et al., 2016). Given the proximity of embryonically derived macrophages to the cardiac vasculature, studies have investigated the potential role of macrophages in heart development beyond a more generalized role in the clearance of senescent cells (Munoz-Espin et al., 2013; Storer et al., 2013). Yolk sac-derived CCR2^−^ macrophages are essential for coronary maturation, facilitating remodeling of the primitive coronary plexus through expansion of the coronary vasculature (Leid et al., 2016). The endocardium is an embryonic source of cardiac macrophages in cardiac valves. Endocardium-derived macrophages are highly phagocytic and indispensable for valve remodeling (Shigeta et al., 2019). Macrophages are also essential for cardiac lymphatic growth and remodeling (Cahill et al., 2021). They closely interact with lymphatic vessels, promoting their growth and fusion during the development of the cardiac vasculature. It is also likely that macrophages play other developmental roles, including in the cardiac conduction system given their involvement in regulating cardiac rhythm in the homeostatic heart (Hulsmans et al., 2017). An interesting phenomenon is that embryos can heal wounds perfectly without scarring (reviewed by Redd et al., 2004), suggesting that macrophages and other resident immune-like cells are programmed differently during development, as distinct from initiating pro-fibrotic repair during adult stages.

### Eosinophils

Eosinophils are innate immune granulocytes best recognized for their effector functions following infection or allergic reactions. They are recruited to the infarcted heart by 24 h post-MI and their numbers peak at 4-days post-MI (Toor et al., 2020). Eosinophils have a cardioprotective function; they reduce cardiomyocyte death, cardiac fibroblast activation and ensuing fibrosis of the post-MI heart. This cardioprotective role is achieved via the production of IL4 (Liu et al., 2020; Toor et al., 2020) (Fig. 1D).

### Innate lymphocytes

Natural killer (NK) cells are innate lymphocytes that infiltrate the heart following MI (Bouchentouf et al., 2010) and play multiple roles to protect the injured tissue. They first limit innate immune cell infiltration and activity through restricting chemokine production and secreting interferon γ (IFNγ), perforin and anti-inflammatory chemokines (Ong et al., 2015). NK cells can also inhibit the overproduction of collagen by myofibroblasts (Fig. 1D) and limit cardiomyocyte apoptosis (Ayach et al., 2006; Ong et al., 2017).

Innate lymphoid cells have recently been identified as a new immune cell subset within the repertoire of innate immune cells activated following cardiac injury. Specifically, innate lymphoid cells type 2 (ILC2) cells form a cluster that secrete type 2 cytokines, such as IL5 and IL13, and are activated through IL25, IL33 and IL2 (Dahlgren et al., 2019; Roediger et al., 2015). ILC2s are a subset of innate lymphocytes that are similar to T cells but cannot express recombined surface antigen receptors, thus functioning in an antigen-independent manner. Perivascular ILC2s regulate macrophages and inhibit atherosclerotic plaque formation (Bracamonte-Baran et al., 2019; Newland et al., 2017). More recently, ILC2 have been shown to reside in pericardial fat, being highly proliferative upon acute MI. The absence of ILC2 in mice leads to increased accumulation of inflammatory monocytes and macrophages in the damaged heart and worsens post-MI fibrotic outcomes. Unraveling the role of ILC2 is a potential novel cellular target to immunomodulatory interventions (Yu et al., 2021).

## Adaptive immune response to heart injury

After the initial innate immune response, activation of the adaptive immune system takes place in the injured heart.

### Dendritic cells

Dendritic cells (DCs) are the major antigen-presenting cells found within the cardiac tissue, some of which localize in cardiac valves (Choi et al., 2009). DCs sample antigens *in situ* and then migrate to the draining lymph nodes where they help generate a population of effector lymphocytes (Fig. 1B). In the injured heart, DCs are crucial for the activation of adaptive, antigen-specific, CD8^+^ (cytotoxic) T lymphocytes in draining mediastinal lymph nodes, establishing an important link between the innate and adaptive immune systems (Dzionek et al., 2001; Forte et al., 2021; Nagai et al., 2014; Satpathy et al., 2012).

### T lymphocytes

**Fig. 2.**
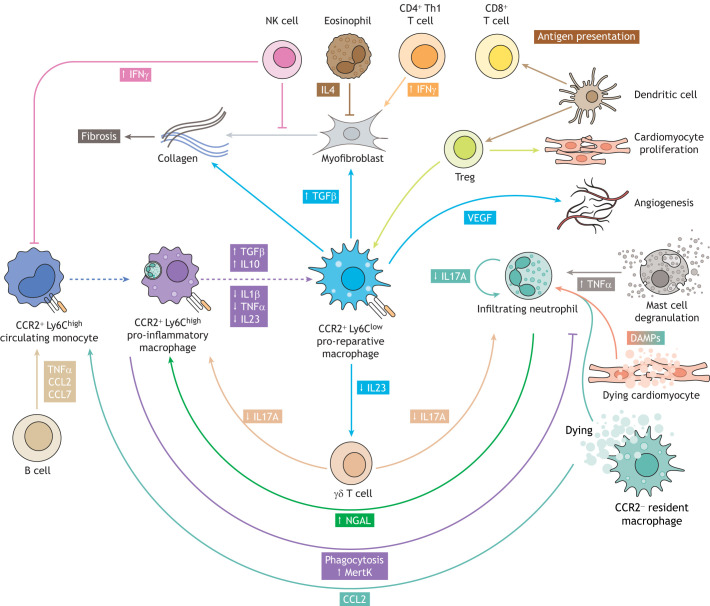
**Immune cell-cell interactions within the damaged heart are crucial for limiting scarring during heart repair.** A key feature in the transition from an inflammatory response to permanent fibrosis or regeneration is the crosstalk between the many different immune cell types that populate the heart. Upon cardiac injury, paracrine signaling occurring between resident cardiac cells (e.g. cardiomyocytes, CCR2^−^ macrophages and mast cells) and infiltrating populations (e.g. neutrophils and CCR2^+^ monocytes and macrophages) determines the kinetics of immune responses and ultimately cardiac output and function following injury. Recruited neutrophils contribute to their own clearance, and therefore resolution of inflammation, by expressing NGAL, a marker that promotes the phagocytic capacity of monocyte-derived CCR2^+^ Ly6C^high^ pro-inflammatory macrophages. Upon ingestion of apoptotic neutrophils, CCR2^+^ Ly6C^high^ monocyte-derived macrophages increase production of anti-inflammatory and pro-fibrotic cytokines (e.g. IL10 and TGFβ) and subsequently downregulate expression of pro-inflammatory cytokines (e.g. IL1β and TNFα). This signaling cascade promotes *in situ* differentiation into CCR2^+^ Ly6C^low^ macrophages and contributes to resolution of tissue inflammation. Phagocytosing macrophages also decrease IL23 expression, which reduces IL17A production by γδ T cells. Whereas γδ T cells and neutrophils release IL17A within the damaged myocardium to increase neutrophil production and recruitment to the injured heart, decreased IL17A expression reduces neutrophil accumulation, which dampens inflammation and improves tissue healing overall. CCR2^+^ Ly6C^low^ pro-reparative macrophages contribute to tissue repair by directly depositing collagen into the fibrotic scar and by activating collagen-depositing myofibroblasts via TGFβ. This pro-fibrotic repair is promoted by CD4^+^ and CD8^+^ T cells through an IFNγ-dependent pathway, but counterbalanced by eosinophil and NK cell inhibition of collagen overproduction by myofibroblasts. Please see main text for further details.

T lymphocytes are formed in the thymus and are programmed to be specific for one given antigen. Once T cells leave the thymus, they bind to the surface of the antigen-presenting cell and become activated. Following activation, circulating CD4^+^ T cells are mobilized to the injured heart and aid myocardial wound healing by promoting CCR2^+^ pro-reparative macrophage activity (Hofmann et al., 2012; Yan et al., 2013) (Fig. 1D). CD4^+^ T cells can be further subdivided into T helper type 1 (Th1), T helper type 2 (Th2) and regulatory T cell (Treg) subsets, according to their phenotype, cytokine production and function. The IFNγ-producing Th1 population plays a detrimental role during MI healing (Yan et al., 2017), supported by reports of a high Th1/Th2 ratio found in post-MI patients with increased adverse cardiac events (Li et al., 2019). Counterbalancing Th1 function, a Foxp3-expressing Treg population (Rudensky, 2011) becomes activated at later stages post-injury and contributes to inflammation resolution by favoring pro-reparative macrophage activation and tissue repair (Saxena et al., 2014; Tang et al., 2012; Weirather et al., 2014; Xia et al., 2020) (Fig. 1D, Fig. 2). Adoptive transfer of Tregs results in a favorable outcome post-MI, with evidence of increased cardiomyocyte proliferation and concomitant reduction in infarct size (Matsumoto et al., 2011; Tang et al., 2012; Zacchigna et al., 2018).

In contrast, CD8^+^ T cells may have both beneficial and detrimental effects on the recovery of the infarcted heart, with their depletion leading to an initial increase in immune cell numbers, but poor scar formation at later stages and ultimately cardiac rupture (Ilatovskaya et al., 2019; Santos-Zas et al., 2021; Tae Yu et al., 2015).

### B lymphocytes

B lymphocytes are adaptive immune cells responsible for mediating the production of antigen-specific immunoglobulin directed against invasive pathogens. B cells infiltrate the injured heart (Yan et al., 2013) and produce cytokines and chemokines, such as CCL2, CCL7 and TNFα (Lund, 2008; Mo et al., 2021; Zouggari et al., 2013), which promote infiltration of circulating CCR2^+^ Ly6C^high^ monocytes and contribute to myocardial remodeling (Porter et al., 2004) (Fig. 1C, Fig. 2). B cell depletion reduces MI size, improving recovery of function, and drug inhibition of B cell infiltration also improves function after acute heart injury (Adamo et al., 2018). Recently, a subset of B cells has been shown to infiltrate the infarcted heart via the CXCL13-CXCR5 axis and contribute to local TGFβ production (Heinrichs et al., 2021).

## Mechanistic targets to limit scarring during heart repair

A key feature in the transition from acute inflammation to permanent fibrosis or regeneration is the crosstalk between the many different cell types that populate the heart. Cell-cell interactions and the resulting paracrine signaling occurring between the various immune cell types determine the kinetics of immune responses, as well as cardiac output and function following injury or disease (Fig. 2).

### Reducing neutrophilic inflammation

One of the first steps for limiting *in situ* tissue damage and promoting the transition to cardiac healing is the resolution of neutrophilic inflammation. Many *in vitro* and *in vivo* studies have shown that the phagocytic capacity of macrophages is key in initiating this neutrophil clearing process (Kaveh et al., 2022; Lai et al., 2017; Lorchner et al., 2015; Schloss et al., 2016; Xu et al., 2019). Depletion of inflammatory macrophages post-MI leads to neutrophil persistence in the damaged area and accumulation of apoptotic material, which exacerbates inflammation and ultimately impairs regeneration (Bevan et al., 2019; de Preux Charles et al., 2016; Lai et al., 2017). After inflammatory Ly6C^high^ monocyte-derived macrophages phagocytose dying neutrophils (Fig. 1D), they increase expression of anti-inflammatory and pro-fibrotic cytokines, such as IL10 and TGFβ, and subsequently reduce the production of IL1β and TNFα pro-inflammatory cytokines (Fadok et al., 1998; Voll et al., 1997; Wan et al., 2013) (Fig. 2). Therefore, upon ingestion of apoptotic neutrophils, Ly6C^high^ monocyte-derived macrophages differentiate into Ly6C^low^ macrophages, contributing to the resolution of tissue inflammation. Phagocytosing macrophages also decrease expression of IL23, which in turn leads to decreased production of IL17A by gamma delta (γδ) T cells, unconventional T cells defined by expression of heterodimeric T cell receptors composed of γ and δ chains (Stark et al., 2005; Tan et al., 2008). γδ T cells and neutrophils release IL17A within the damaged myocardium, which feeds back to the bone marrow to increase neutrophil production and recruitment to the injured heart. Therefore, decreased IL23 expression by phagocytosing macrophages leads to lower IL17A levels in cardiac tissue, reducing neutrophil accumulation *in situ*, dampening inflammation and improving tissue healing (Yan et al., 2012) (Fig. 2).

### Modulating dendritic cell-mediated T cell activation

As a result of cardiomyocyte death, the major sarcomeric structural protein alpha-myosin heavy chain (αMyHC) is released in the heart. Activated DCs, mainly conventional type 2 DCs (cDC2s), migrate to the lymph node and present αMyHC to T cells (Van der Borght et al., 2017). This is problematic because, during development, CD4^+^ T cells specific for cardiac self-antigen αMyHC escape thymic negative selection (Lv et al., 2011). Therefore, αMyHC release by the infarcted heart is perceived as non-self, such that antigen presentation by cDC2s induces Th1/Th17 autoreactive T cells. Such an autoimmune response against major cardiac proteins is commonly observed in MI patients (Moraru et al., 2006), usually contributing to persistent inflammation, further cardiac damage and heart failure (Gottumukkala et al., 2012). However, non-selective depletion of all DCs to avoid autoactivation and loss-of-tolerance is not beneficial, because this leads to increased infiltration of Ly6C^high^ monocytes and impaired differentiation of Ly6C^low^ macrophages to the injured heart, sustaining an inflammatory state of the damaged tissue (Anzai et al., 2012; Choo et al., 2017; Shiraishi et al., 2016). Interestingly, when only the conventional DC subpopulation is impaired, lower numbers of macrophages, neutrophils and T cells are present at the injury site, which consequently reduces chronic inflammation and fibrosis, preventing adverse cardiac remodeling (Lee et al., 2018). Furthermore, injection of tolerogenic DCs (DCs with immunosuppressive properties) into the damaged heart induces the activation of MI-specific Tregs in mediastinal lymph nodes, which promote a phenotypic macrophage shift from pro-inflammatory to pro-reparative states (Choo et al., 2017) (Fig. 2). Therefore, although DCs can exacerbate chronic outcome through loss of tolerance, they also promote tissue healing and act as immunoregulators through the recruitment and activation of other immune cell types located outside the damaged heart.

### Interactions with non-immune cells for cardiac repair

Although hematopoietic immune cells are the bona fide components of the immune system, immune functions are not unique to hematopoietic cells; other cell types display basic mechanisms of immunity (Arinda et al., 2022; Guerrero-Juarez et al., 2019; Kinchen et al., 2018; Mizoguchi et al., 2018; Rodda et al., 2018). Recently, the term ‘structural immunity’ has been proposed for the study of immune functions in non-hematopoietic cell populations. It has been applied to three major cardiovascular cell types: epithelium, endothelium and fibroblasts, which act as key regulators of organ-specific immune responses (Krausgruber et al., 2020). There are also examples where communication of immune cells and non-immune resident cardiac cells influence the post-injury response. For example, non-immune resident cardiac nerves release neuropeptide substance P and neurotensin, which induces mast cells to promote cardiac remodeling via histamine secretion (Melendez et al., 2011; Pang et al., 1998).

As mentioned above, during the pro-reparative phase after heart injury, macrophages directly contribute to ECM deposition and indirectly help repair the damaged tissue by interacting and activating resident cardiac fibroblasts via TGFβ (Simoes et al., 2020; Swirski and Nahrendorf, 2018). Activation of cardiac fibroblasts into myofibroblasts is also promoted by CD4^+^ and CD8^+^ T cells through an IFNγ-dependent pathway (Marko et al., 2012) (Fig. 2).

During the reparative phase, CCR2^+^ Ly6C^low^ reparative macrophages can also contribute to angiogenesis (Frangogiannis, 2012; Hilgendorf et al., 2014), while Tregs promote cardiomyocyte proliferation (Hui et al., 2017) (Fig. 2). In addition, NK cells have protective effects on the damaged heart by physically interacting with endothelial cells to promote angiogenesis (Bouchentouf et al., 2010). Furthermore, crosstalk between lymphatic endothelial cells and immune cells can improve cardiac function post-MI (Henri et al., 2016; Houssari et al., 2020; Vieira et al., 2018). Cardiac lymphatics secrete chemokines (e.g. CCL21, CX3CL1 and CXCL12), which attract specific populations of immune cells according to their receptor expression profile. VEGFC-driven increase in lymphangiogenesis also improves cardiac function after infarction by increasing immune cell clearance through an LYVE1-dependent mechanism (Houssari et al., 2020; Vieira et al., 2018).

## A regenerative immune response

The damage caused by a heart attack cannot be repaired in adult mammals, leading to permanent loss of cardiac tissue. In contrast, organisms such as zebrafish, axolotl and *Astyanax mexicanus* surface fish have remarkable regenerative capacity across organ systems, including the heart. Each of these animals presents an opportunity to investigate how evolution has retained natural regeneration in these species and to understand what has potentially been lost or adapted in adult mammals. Effective cardiac regeneration depends not only on the degree of regenerative competence of the injured heart (Jopling et al., 2010; Kikuchi et al., 2010; Porrello et al., 2011), but also on the transient nature of the fibrotic-based repair (Bevan et al., 2019; Sanchez-Iranzo et al., 2018; Simoes et al., 2020). The transition from a reparative phase to a regenerative phase following heart injury requires an orchestrated immune response to fine-tune the interplay between a pro-fibrotic and a pro-regenerative environment (Aurora et al., 2014; Bevan et al., 2019; Lai et al., 2017; Sanz-Morejon et al., 2019) (Fig. 1F).

### Zebrafish

In the zebrafish, various studies have now described a robust and dynamic activation of innate and adaptive immune cell subsets in the injured zebrafish heart (reviewed in detail by Ryan et al., 2020). Such studies have revealed evolutionarily conserved features of the post-injury immune response, which is sufficiently robust to argue against the idea that the capacity for tissue regeneration is inversely correlated with the evolutionary complexity of the immune system (Mescher and Neff, 2005). Rather, it is the type of immune response that is key to dictating a successful regenerative outcome after injury. Comparing the molecular programs deployed by macrophages in the post-resection zebrafish heart, which completes regeneration quickly and without scar formation (Poss et al., 2002), and the cryoinjured heart, which shows a delay in the regenerative process and forms a transient fibrotic scar (Chablais et al., 2011; Gonzalez-Rosa et al., 2011; Schnabel et al., 2011), has revealed crucial differences in the kinetics of the innate immune response to different post-injury environments (Simoes et al., 2020). A synchronized pro- and anti-inflammatory response are coincident with the rapid regenerative process occurring in the scar-free resected heart. Conversely, in the scar-inducing heart, an initial pro-inflammatory response is replaced by anti-inflammatory macrophages, preventing permanent fibrosis and allowing for effective regeneration (Simoes et al., 2020). Further dissection into macrophage subpopulations, including how these subpopulations shift or emerge according to different post-injury environments and wound-healing kinetics, could ultimately inform on which subpopulations to target to modulate excessive inflammation or pro-fibrotic tissue repair upon injury.

### Axolotls

Axolotls present most of the components of the human immune system (Godwin et al., 2013; Lopez et al., 2014; Ussing and Rosenkilde, 1995). Characterization of the immune response following cardiac injury has revealed that eosinophils, neutrophils, monocytes, lymphocytes and plasma cells are activated and recruited to the site of injury. Macrophage ablation reduces the regenerative capacity of the axolotl cryoinjured heart (Cano-Martinez et al., 2010; Godwin et al., 2017), leading to impaired neovascularization, decreased fibroblast activation and compromised heart function, despite successful cardiomyocyte proliferation. These findings have revealed that the cause of regenerative failure in macrophage-depleted cryoinjured axolotls is not dependent on cardiomyocytes re-entering the cell cycle. Rather, macrophage depletion during the early stages post-injury modifies cardiac ECM synthesis, remodeling and cross-linking, which collectively blocks regeneration. Therefore, in the axolotl, although cardiomyocyte proliferation is necessary for effective regeneration, it is not sufficient to prevent fibrotic progression. Further deciphering of the mechanism by which macrophage-fibroblast interactions appear to repress fibrotic activation in the axolotl heart may lead to new molecular targets to optimize repair in the mammalian heart.

### Astyanax mexicanus

*Astyanax mexicanus* cavefish and surface fish respond differently to cardiac injury. Although they are the same species, surface fish can regenerate their hearts after injury, whereas the cave-dwelling fish cannot, instead forming a permanent fibrotic scar (Stockdale et al., 2018). Upregulation of both immune and scarring responses has been reported in injured non-regenerative cavefish hearts when compared with regenerating surface fish counterparts (Stockdale et al., 2018). Similar comparative studies in zebrafish and the non-regenerative medaka (Japanese rice fish) have shown the importance of a timely and robust activation, and subsequent resolution, of the immune response to injury for successful cardiac regeneration (Lai et al., 2017). Therefore, it is important to further dissect the kinetics and cellular phenotypes of the immune response in regenerative versus non-regenerative fish species to understand the crucial nature and timing of the immune response in achieving tissue regeneration.

### Neonatal mammals

Although the adult mammalian heart cannot regenerate upon injury, the neonatal mouse heart can, but only during the first 7 days after birth (Porrello et al., 2011). Macrophages are directly implicated in mediating this regenerative response, in part through the expression of growth factors, such as IGF1 (Aurora et al., 2014). Global macrophage depletion leads to a loss of cardiac regenerative capacity, with deposition of a permanent scar, impaired neovascularization and reduced cardiac function (Aurora et al., 2014). However, selective depletion of tissue-resident CCR2^−^ macrophages reduces proliferation of cardiomyocytes and endothelial cells and increases interstitial fibrosis in the neonate injured heart (Lavine et al., 2014). Following neonatal heart injury, the number of tissue-resident CCR2^−^ macrophages expands without the additional infiltration of CCR2^+^ monocytes, which is very different from what happens in the injured adult heart (Lavine et al., 2014). Therefore, it is tempting to speculate that the limited capacity of the neonatal heart to recruit infiltrating monocytes in large numbers following injury may be an intrinsic mechanism that promotes regenerative programs associated with a prevalence of resident, embryonic-derived CCR2^−^ macrophages. In addition, the transcriptional response of macrophages in the neonate heart is dampened, relative to the highly expressed inflammatory and reparative transcriptional signatures observed in macrophages of the adult post-MI heart (Simoes et al., 2020). These studies collectively suggest that the distinct transcriptional programs underlying macrophage function during neonate and adult mouse cardiac injury may contribute to the transition from regenerative to scar-based repair.

## Recent technological advances to probe the immune response to heart injury

A fundamental roadblock to deciphering the dual role of immune cells in promoting injury and repair of the damaged heart is the lack of a comprehensive understanding of their phenotypic and functional diversity.

Historically, high-throughput bulk transcriptional profiling of the injured heart has been performed on populations of immune cells isolated from the heart by fluorescence-activated cell sorting (FACS), based on marker gene expression. However, discriminating state and function within bulk RNA-sequencing of mixed immune cell populations is not possible due to their inherent heterogeneity (Dick et al., 2019). Recent technological advances in single-cell transcriptomics have greatly refined our functional understanding of immune cell subsets (Depuydt et al., 2020; Dick et al., 2019; Fernandez et al., 2019; Molenaar et al., 2021; Vafadarnejad et al., 2020; Vallejo et al., 2021 preprint; Wirka et al., 2019). For example, a combination of single-cell RNA-sequencing (scRNA-seq) and genetic fate mapping has revealed four subpopulations of cardiac macrophages in the homeostatic adult mouse heart, which further diversify upon cardiac injury. This study uncovered TIMD4, a phosphatidylserine receptor, and CCR2 as mutually exclusive markers of resident versus recruited macrophages in the post-MI heart (Dick et al., 2019).

When unbiased scRNA-seq is combined with immunophenotyping of cells using antibody sequencing (CITE-seq) (Stoeckius et al., 2017), the resolution of immune cell diversity is even greater. The integration of scRNA-seq and CITE-seq datasets of circulating monocytes and cardiac macrophages following MI has identified new markers that can be used to track specific monocyte and macrophage subpopulations in the mouse heart (Rizzo et al., 2020 preprint). Notably, this study suggests that infiltrated monocytes develop into two main post-MI macrophage subpopulations: Trem2^hi^Igf1^hi^ and MHCII^+^ macrophages. It also proposes that MGL2 and CD81 are two surface markers that may help distinguish resident macrophage populations from monocyte-derived macrophages in the injured heart. One caveat with scRNA-seq approaches to identify immune cell subtypes is the reliance on existing marker sets to adequately cluster cells that are, by definition, biased (dependent upon previous studies and identification of candidate markers) and may not be entirely accurate to attribute function. Employing broad-based initial cell isolation (e.g. using CD45^+^ to capture all leucocytes) enables a more unbiased approach, which computational analysis may cluster into cell subtypes more accurately. Ultimately, by linking both the transcriptome and the proteome, new and reliable cell surface markers may be identified that enable purification and localization of rare immune cell subsets by flow cytometry and confocal microscopy.

Single-cell technologies can profile the molecular heterogeneity of the different immune cell types; however, without spatial context, it is unclear how various immune cells coordinate their function within the injured heart. The advent of spatial transcriptomics is revolutionizing the way we understand tissue biology in homeostasis and disease (Asp et al., 2019; Marx, 2021). Currently, spatial transcriptomics lacks bona fide cellular resolution; regardless, the evolving platforms can identify discrete cell clusters in a spatial context, which, when integrated with insight into single-cell gene expression profiles, provides significant insights into cellular crosstalk and potential interactions within the local injury environment. Recently, the integration of spatial transcriptomics, single-cell gene expression and chromatin accessibility of the human infarcted heart has provided unprecedented insights across the various cell types residing in the heart and their response to injury (Kuppe et al., 2020 preprint). This study is a valuable resource for further investigation into the location of specific immune cell subpopulations relative to the site of injury and the potential cell-cell signaling that might be essential for their function. The International Human Cell Atlas initiative has facilitated the collection of single-cell transcriptomic data of the human heart and has identified eight populations of lymphoid cells and 13 populations of myeloid cells, including multiple subtypes of macrophages, monocytes and dendritic cells (Litvinukova et al., 2020). These findings are informed by the first studies on the human immune response post-MI, which indicate that similar monocyte and macrophage subsets are involved in the mouse and human heart (Bajpai et al., 2018; Chen et al., 2021; Ruparelia et al., 2015; van der Laan et al., 2014). Comparing these cells and their molecular signatures with knowledge gained from animal models will be important to identify predictive biomarkers for disease progression, for the early identification of patients at risk of heart failure, and/or finding biomarkers for pro-regenerative immune cell subsets.

A holistic view of the spatial-temporal heterogeneity of the various immune cells responding to heart injury will allow us to link (epi)genomic diversity to changes in cellular function. Options to selectively modulate immune cell activity have increased dramatically over the past few years. Just a few examples include CRISPR-Cas9 genome editing, pharmacological targeting and nanoparticle delivery of therapeutic cargo; including the latest developments on lipid nanoparticles loaded with mRNA technology further developed and validated during the COVID-19 pandemic (Hou et al., 2021). All these technologies will undoubtedly contribute to better define the functional diversity of specific immune subsets in the injured heart.

## Conclusion

The immune response to cardiac injury is multifaceted. A deeper understanding of how and when immune cell types orchestrate inflammatory and reparative pathways following cardiac injury will lead to effective therapeutic strategies that promote tissue regeneration and improve recovery in post-MI patients. Thus far, studies focusing on macrophages have dominated the landscape. This is understandable given their evolving plasticity in terms of diverse subtypes and function, as well as the fact they are the dominant immune cells in terms of sheer numbers that present following MI. That said, other cell types are very important and have profound effects on both repair and regeneration, as described above. Neutrophils fall into this category, as initial responders within the innate immune spectrum. The role of adaptive immunity is only just being explored in terms of effects on acute injury, chronic disease progression and the development of heart failure. As such, DCs, T cells and B cells require further study to provide basic insight into how these various immune cells interact both with one another and with non-immune cardiovascular cells. A deeper understanding of innate and adaptive immune cell interactions, across both acute and chronic stages of injury, may provide potential therapeutic targets to modify outcome post-MI.

To date, many of the immune modifying therapies have tested immunosuppressive drugs that bluntly inhibit inflammation (D'Amario et al., 2021; Deftereos et al., 2014; Huang and Frangogiannis, 2018; Schmidt et al., 2016; Verma et al., 2015). This approach presents challenges because an initial pro-inflammatory response is essential for the clean-up operation in the heart after ischemic cell death and feeds forward to effective cardiac repair. In addition, many of the immune functions that are harmful to the heart are necessary for host defense, leaving immune-suppressed patients highly vulnerable to infections and viral attack (Huang and Frangogiannis, 2018). Most of the clinical trials to date have led to disappointing outcomes in terms of enhancing cardiac repair after injury, with some even having a negative impact. For example, targeted disruption of the complement cascade (Armstrong and Granger, 2007), TNFα blockade (Mann et al., 2004) and delivery of CXCL12 (Chung et al., 2015) have not yielded cardiac functional improvements, whereas administration of corticosteroids (Huang and Frangogiannis, 2018; Schmidt et al., 2016) and colchicine (D'Amario et al., 2021; Deftereos et al., 2014; Verma et al., 2015) increase incidence of cardiac rupture. The challenge is to selectively modulate the immune system in a way that specifically targets components of the response to reduce excessive inflammation and favor reparative functions. Although transcriptional and proteomic profiling of single cells has enabled reconstruction of stable cellular identities, transitions between cellular states in highly dynamic scenarios have been much harder to characterize. 4D live imaging has highly complemented these technologies, providing behavioral landscapes during acute inflammatory responses, which are characterized by continuous changes in the motility and morphology of individual cells as they adapt to ever-changing local cues (Crainiciuc et al., 2022; Kaveh et al., 2020; Taylor et al., 2019). Recent efforts on targeting immunomodulators include inhibition of the coagulation cascade (Gadi et al., 2021) and IL4 delivery (Han et al., 2018), which shift the balance of macrophage response towards a reparative phenotype by reducing harmful inflammation and have delivered more beneficial outcomes in acute MI patients. Blocking specific cytokines, such as IL1β, via monoclonal antibody targeting (Canakinumab; CANTOS trial) (Abbate et al., 2010; Everett et al., 2019) reduces post-MI heart failure and delivers beneficial effects in atherosclerotic disease (Ridker et al., 2017). These promising results reinforce the idea that dampening the inflammatory response via targeted immune modulators may significantly enhance the treatment of cardiovascular disease. Looking ahead, advances in chimeric antigen receptors engineered into cytotoxic T cells (CAR T cells) offer exciting opportunities to improve cardiac repair. Taking advantage of the distinctive cell surface markers expressed by activated cardiac fibroblasts (Kaur et al., 2016), which are sufficiently different from the ones presented by quiescent fibroblasts, CAR T cells have been specifically designed to target active fibroblasts and reduce cardiac fibrosis in the mouse heart (Aghajanian et al., 2019; Rurik et al., 2022). Given how successfully CAR T cells have been adapted to successfully treat many blood-associated cancers (Feins et al., 2019), these results provide a robust proof-of-concept for the future use of engineered T cells to reduce cardiac fibrosis in post-MI patients.
